# One amino acid makes a difference–Characterization of a new TPMT allele and the influence of SAM on TPMT stability

**DOI:** 10.1038/srep46428

**Published:** 2017-05-02

**Authors:** Yan Ping Heidi Iu, Sara Helander, Anna Zimdahl Kahlin, Chun Wah Cheng, Chi Chung Shek, Moon Ho Leung, Björn Wallner, Lars-Göran Mårtensson, Malin Lindqvist Appell

**Affiliations:** 1 Chemical Pathology Laboratory, Department of Pathology, Queen Elizabeth Hospital, Hong Kong SAR, China; 2 Division of Drug Research, Department of Medical and Health Sciences, Linköping University, SE-581 85 Linköping, Sweden; 3 Department of Medicine, Queen Elizabeth Hospital, Hong Kong SAR, China; 4 Division of Bioinformatics, Department of Physics, Chemistry and Biology, Linköping University, 58183 Linköping, Sweden; 5 Division of Chemistry, Department of Physics, Chemistry and Biology, Linköping University, SE-581 83 Linköping, Sweden

## Abstract

Thiopurine induced toxicity is associated with defects in the thiopurine methyltransferase (TPMT) gene. TPMT is a polymorphic enzyme, with most of the single nucleotide polymorphisms (SNPs) causing an amino acid change, altering the enzymatic activity of the TPMT protein. In this study, we characterize a novel patient allele c.719A > C, named TPMT*41, together with the more common variant *3C c.719A > G, resulting in an amino acid shift at tyrosine 240 to serine, p.Y240S and cysteine, p.Y240C respectively. We show that the patient heterozygote for c.719A > C has intermediate enzymatic activity in red blood cells. Furthermore, *in vitro* studies, using recombinant protein, show that TPMT p.Y240S is less stable than both TPMTwt and TPMT p.Y240C. The addition of SAM increases the stability and, in agreement with Isothermal Titration Calorimetry (ITC) data, higher molar excess of SAM is needed in order to stabilize TPMT p.Y240C and TPMT p.Y240S compared to TPMTwt. Molecular dynamics simulations show that the loss of interactions is most severe for Y240S, which agrees with the thermal stability of the mutations. In conclusion, our study shows that SAM increases the stability of TPMT and that changing only one amino acid can have a dramatic effect on TPMT stability and activity.

Individual drug dose responses due to genetic variations are a well-known problem and pharmacogenomics studies are a key factor in the work towards personalized drug treatment. The human enzyme thiopurine S-methyltransferase (TPMT) is involved in the metabolic pathways of thiopurine drugs, such as azathiopurine (AZA) and 6-mercaptopurine (6-MP), used to treat acute lymphoblastic leukemia (ALL), inflammatory bowel diseases (IBD) and rheumatological diseases^1^,^2^. Genetic variations in the TPMT gene accounts for many of the drug related side effects associated with thiopurine drugs and although the treatment of ALL is a success in many respects, up to 25% of children suffer from severe side effects, due to treatment with 6-MP^3^. In addition, achieving optimal thiopurine treatment for IBD patients is difficult and around 40% of IBD patients show an adverse response to the thiopurine therapy, either by suffering from adverse side effects or by being resistant to therapy^4^,^5^,^6^,^7^. The normal metabolic pathway involves transformation of AZA and 6-MP to methylmercaptopurine (meMP), by the transfer of a methyl group from the TPMT co-factor S-adenosylmethionine (SAM) by TPMT, thereby reducing the level of active thioguanine nucleotides (TGNs) that can be incorporated in DNA. Drug induced toxicity is associated with defects in the TPMT gene, resulting in an altered enzymatic activity for the TPMT protein, with increased incorporation of TGNs into DNA as a result of the altered activity^8^. Since 1980 it has been known that TPMT is a polymorphic enzyme and so far around 40 allelic variants of the TPMT enzyme have been discovered, most of which are single nucleotide polymorphisms (SNPs) causing an amino acid change^8^,^9^,^10^ (https://www.imh.liu.se/tpmtalleles). Approximately 10% of the Swedish population and 5% of the Chinese population carries SNPs in the gene for TPMT, which in most cases results in decreased enzymatic activity of the TPMT protein^11^,^12^. The distribution of alleles varies among different populations; most common among the European population are TPMT*2, TPMT*3A and TPMT*3C. Among them, the SNP for TPMT*3C at exon 10, c.719A > G is one of the most common alleles in both European and Asian populations^13^,^14^,^15^. The SNP c.719A > G results in an amino acid (a.a) substitution at position 240 from Tyr to Cys^16^,^17^. Tyr240 is highly conserved, fully exposed in the 3D structure and located in an aggregation prone region in the β9-sheet at the C-terminal end of the protein^18^,^19^. The network of molecular hydrophobic and polar interactions surrounding Tyr240 form a hydrophobic core essential for protein stability and function, and variations at this residue will result in altered protein stability^19^.

Recently, genome wide association studies (GWAS) have emphasised polymorphic variation in TPMT as the principle determinant of TPMT activity^20^,^21^. However, the finding that polymorphisms in the methylene tetrahydrofolate reductase (MTHFR) gene could indirectly influence TPMT activity^22^, by altering intracellular SAM levels as a result of the affected folate metabolism, leads to the question of whether SAM could function as a predictor of the enzymatic activity. By studying SAM levels and TPMT activity in a leukemic cell line, it has been shown that adding exogenous SAM to culturing media could sustain the intracellular levels of adenosine triphosphate (ATP) and SAM and delay the cytotoxic mechanisms, induced by 6-MP^23^. Earlier studies comparing the intrinsic stability, at 37 °C, of TPMT*1, TPMT*3B and TPMT*3C revealed that, despite the fact that no loss of immunodetectable protein occurred, the addition of SAM partially stabilized the catalytic activity of TPMT*3C^24^. Furthermore, in a recent study evaluating the genotype and phenotype for 1017 healthy blood donors not treated with thiopurine drugs, only the TPMT genotype and SAM levels in red blood cells (RBC) were found to influence TPMT activity^25^. The correlation between TPMT genotype and SAM levels is further emphasized by the fact that heterozygous TPMT*1/*3 individuals with high SAM levels show a more pronounced enzymatic effect compared to individuals with low SAM levels^25^.

To gain further insight into the possible stabilizing effect of SAM on TPMT, it is valuable to address this question on a more detailed level, by studying this process using biophysical methods. To date, no studies have been carried out using recombinant protein and studying the direct effect of SAM on TPMT.

In this study, we use biophysical methods to study the stabilizing effect of SAM on TPMT. In addition, we characterize and study the effect of SAM on a novel patient allele, named TPMT*41, comprising SNP c.719A > C, which results in an amino acid change at a.a 240 from Tyr to Ser. Since we use recombinant protein to study TPMT, we will from now on refer to TPMT*3C as TPMT p.Y240C and the novel TPMT*41 as TPMT p.Y240S when discussing the various proteins. TPMT*1 is defined as TPMTwt (https://www.imh.liu.se/tpmtalleles).

We demonstrate that an individual heterozygous for TPMT p.Y240S has intermediate enzymatic activity in red blood cells, comparable with TPMT p.Y240C. Thus, the biophysical properties for TPMTwt versus TPMT p.Y240C and TPMT p.Y240S differ. In this study, we evaluated the thermal stability and show that TPMT p.Y240S has lower thermal stability than both TPMTwt and TPMT p.Y240C. The addition of SAM increases the stability and, in agreement with Isothermal Titration Calorimetry (ITC) data, higher molar excess of SAM is needed in order to stabilize TPMT p.Y240C and TPMT p.Y240S compared to TPMTwt. Furthermore, by measuring the enzymatic activity on recombinant protein we could conclude, in agreement with thermal stability data, that TPMT p.Y240S shows a remarkably decreased enzymatic activity compared to TPMTwt and TPMT p.Y240C. Molecular dynamics simulations show that the loss of interactions is most severe for Y240S, which agrees with the thermal stability of the mutations.

## Results

### *In vivo* data of c.719A > C

**Figure 1:**
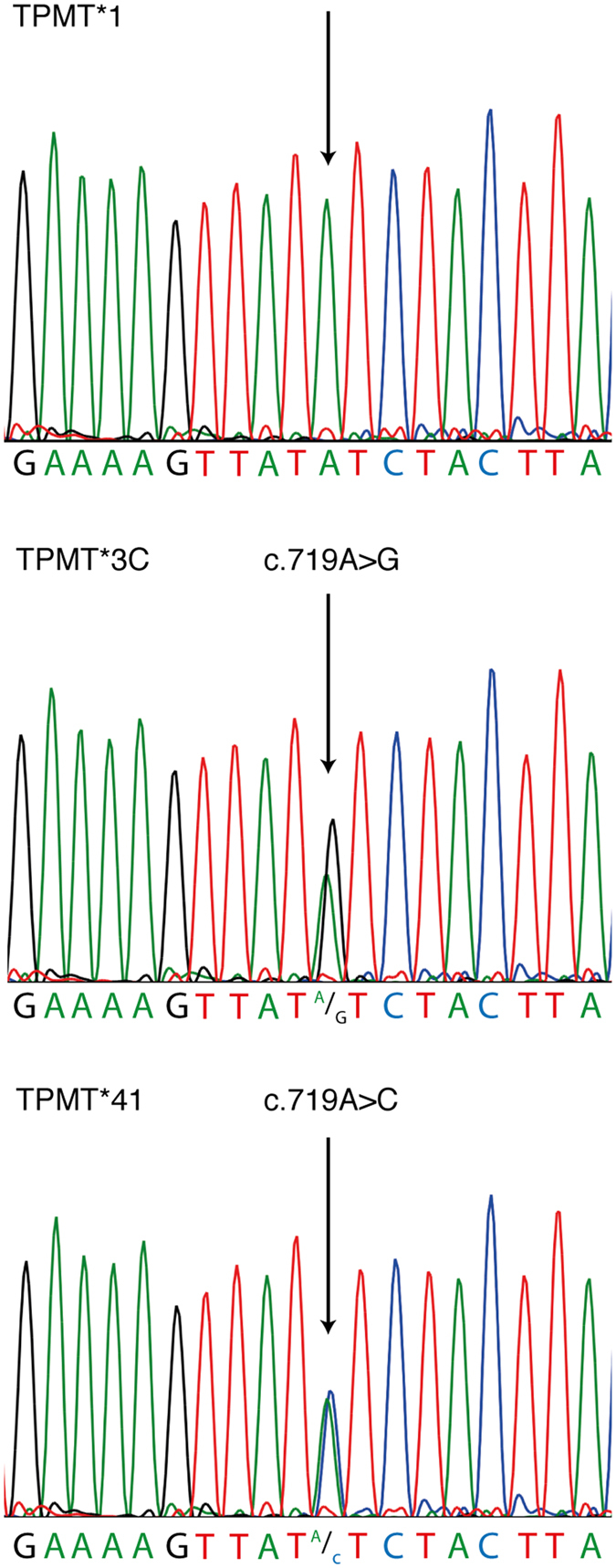
Sequencing histogram from TPMT*1, TPMT*3C and TPMT*41, showing the normal sequence for TPMT*1 and the detected SNP (black arrow) for TPMT*3C (c.719A > G) and TPMT*41 (c.719A > C).

The patient’s TPMT enzyme activity in RBC was 7.7 U/ml pRBC (mean value of two measurements, both within the reference value of intermediate TPMT activity 2.5–9 U/ml pRBC^26^). A novel heterozygous SNP in position c.719A > C identified by sequencing fulfilled the inclusion criteria of the TPMT Nomenclature Committee for novel alleles^10^ and was named TPMT*41 c.719A > C (Fig. 1), the presence of this SNP was further confirmed by pyrosequencing analysis (data not shown). TPMT exon 2–10 was sequenced with only c.719A > C detected deviating from the wildtype sequence. TPMT c.719A > C causes an amino acid shift of p.Y240S.

### Pathogenicity prediction

The tolerance for amino acid substitution of the novel variant, TPMT p.Y240S as well as TPMT p.Y240C, was predicted using three types of *in silico* software: SIFT, PolyPhen-2 and AlignGVGD^27^,^28^,^29^. The SIFT analysis predicted the substitution from Tyr to Cys as deleterious for the protein with a score of 0.04. PolyPhen-2 predicted both substitutions as probably damaging with a score of 1.000. Finally, AlignGVGD predicted both substitution as deleterious 2.

### TPMT p.Y240S is less stable than both TPMTwt and TPMT p.Y240C

**Figure 2:**
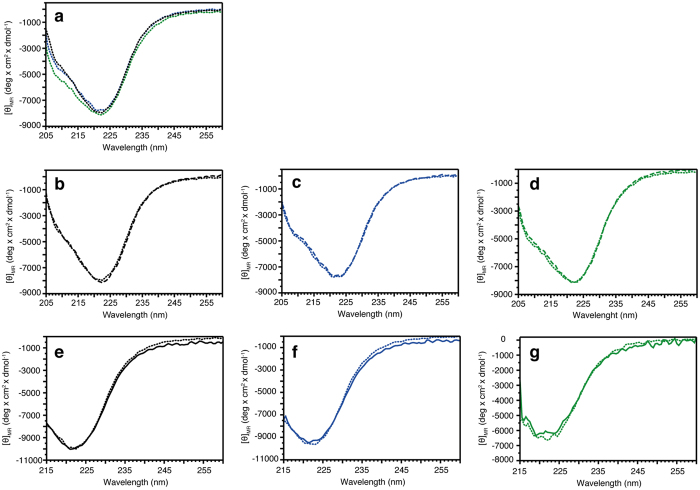
Far-UV spectra of TPMTwt (black), TPMT p.Y240C (blue) and TPMT p.Y240S (green) in absence or presence of 10-fold or 50-fold molar excess of SAM. (**a**) Absence of SAM. (**b–d**) Absence (dotted line) or 10-fold molar excess (dashed line). (**e–g**) Absence (dotted line) or 50-fold molar excess (solid line).

**Figure 3:**
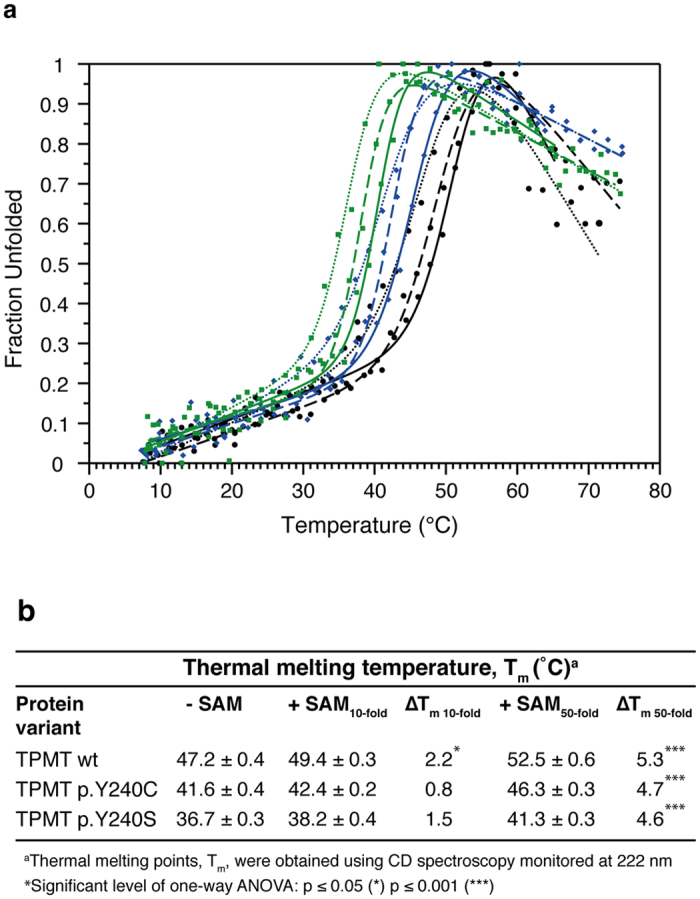
(**a**) Thermal stability of TPMTwt (black), TPMT p.Y240C (blue) and TPMT p.Y240S (green) in 10-fold (dashed line), 50-fold (solid line) molar excess of SAM or absence (dotted line) of SAM. Data from one representative measurement of each protein sample are shown in the graph. (**b**) Table showing thermal denaturation temperatures for TPMTwt, TPMT p.Y240C and TPMT p.Y240S. The thermal denaturation temperatures shown are determined by using the CDpal software^45^ and merging all data points from each individual measurement. Data were fitted to the merged points using a two-state denaturation model.

In order to evaluate the secondary structure and thermal stability, we recorded circular dichroism (CD) spectra for all proteins both in absence and in presence of 10- or 50-fold molar excess of SAM. In agreement with previous studies on TPMTwt protein and TPMT p.Y240C protein^30^, no major differences between the secondary structures for TPMTwt, TPMT p.Y240C and TPMT p.Y240S could be observed and the global secondary structures were not affected by the addition of either 10 or 50-fold molar excess of SAM (Fig. 2). Further, we analyzed the thermal unfolding process and changes in secondary structure content at 222 nm (Fig. 3), where helical unfolding is best followed. The results show that both proteins TPMT p.Y240C and TPMT p.Y240S are less stable compared to TPMTwt (Fig. 3), with thermal midpoint of denaturation (T_m_) temperatures of 41.6, 36.7 and 47.2 °C, respectively.

### SAM increases the thermal midpoint of denaturation temperatures

In presence of 10-molar excess of SAM, all three proteins show a slight increase in stability (Fig. 3). Although the ΔT_m_ at 10-fold molar excess is moderate for TPMTwt, the small increase is significant (p-value ≤ 0.05), while the ΔT_m_ is not significant for TPMT p.Y240C and TPMT p.Y240S (Fig. 3b). Since all protein variants showed a slightly increased ΔT_m_ at 10-fold molar excess of SAM, but a significant increase could be observed only for TPMTwt, our results indicate that TPMT p.Y240C and TPMT p.Y240S have weaker affinity to SAM compared to TPMTwt. We performed additional measurements using 50-fold molar excess of SAM. The results from the additional measurement clearly showed that higher concentrations of SAM were needed in order to stabilize TPMT p.Y240C and TPMT p.Y240S (p-value ≤ 0.001) (Fig. 3). Thus, to investigate this further, we analyzed the thermal stability at 37 °C by monitoring the thermal unfolding at 222 nm during a time period of 8 hours (Table 1). In agreement with our previous measurements, the results showed that TPMTwt is most affected by the addition of 10-fold molar excess of SAM, while TPMT p.Y240C and TPMT p.Y240S were unaffected at the same SAM concentration. For all samples, a decrease in CD signal at 222 nm is seen during the first 30 minutes, indicating an initial unfolding process. Thus for TPMTwt and TPMT p.Y240C in absence of SAM, the signal continuously decreased during the 8 hour measurement. This was in contrast to TPMT p.Y240S, where the CD signal was stabilized at a constant level after the first initial decrease. For TPMTwt, the addition of both 10- and 50-fold molar excess of SAM affected the unfolding process, possibly by stabilizing the protein, and the signal was significantly (p-value ≤ 0.001) stabilized at a constant level (Table 1). In comparison, the signal for TPMT p.Y240C and TPMT p.Y240S was unaffected by the addition of 10-fold molar excess of SAM (Table 1). The addition of 50-fold molar excess of SAM significantly affected the residual CD signal for TPMT p.Y240C (p-value ≤ 0.001), meaning that the long time stability at 37 °C TPMT p.Y240C was increased by the addition of high SAM concentrations. For TPMT p.Y240S, no significant stabilization was observed upon addition of 50-fold molar excess of SAM.

Although the possible binding of SAM to TPMTwt, TPMT p.Y240C and TPMT p.Y240S needs to be evaluated further, all CD measurements showed that both TPMT p.Y240C and TPMT p.Y240S were less stable compared to TPMTwt. Also, compared to TPMTwt, higher SAM concentrations were needed in order to stabilize TPMT p.Y240C and TPMT p.Y240S.

### The enzymatic activity of TPMTwt, p.Y240C and p.Y240S are in agreement with thermal stability data

**Figure 4:**
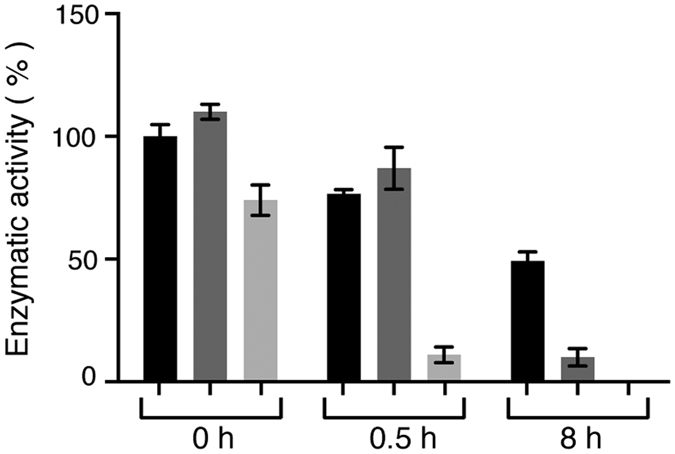
Enzymatic activity of TPMTwt (black), TPMT p.Y240C (dark grey) and TPMT p.Y240S (light grey) at 37 °C after 0 h, 0.5 h or 8 h incubation at 37 °C. The relative enzymatic activity was calculated as a percentage of TPMTwt enzymatic activity at time point 0 h and visualized as % of TPMTwt and mean ±SEM (N = 3).

In order to further characterize the proteins we conducted enzymatic activity measurements following incubation at 37 °C on recombinant proteins TPMTwt, TPMT p.Y240C and TPMT p.Y240S. In agreement with thermal stability data, showing a 10.5 °C reduced ΔT_m_ for TPMT p.Y240S compared to TPMTwt, TPMT p.Y240S displayed lower enzymatic activity compared to TPMTwt (Fig. 4). We could conclude that the change in enzymatic activity was significantly reduced for all proteins after 0.5 h and 8 h (p-value ≤ 0.001). In addition, when comparing TPMT p.Y240C and TPMT p.Y240S at each time point (0 h, 0.5 h and 8 h), the enzymatic activity was changed compared to TPMTwt at the same time point (p-value ≤ 0.001, Fig. 4). At the start (time point 0 h), the enzymatic activity of TPMT p.Y240S was decreased compared to TPMTwt. TPMT p.Y240C displayed a higher activity, compared to TPMTwt at the same time point. The intra assay coefficient of variation was calculated to approximately 5%, which in part explains the higher activity of TPMT p.Y240C. For samples preincubated for 0.5 h, TPMTwt and TPMT p.Y240S displayed lower activity compared to TPMTwt at time point zero. Thus, at the same time point, TPMT p.Y240S showed a dramatic decrease in activity. After 8 hours of incubation time, the activity for TPMTwt had decreased to 49% while TPMT p.Y240C and TPMT p.Y240S only had 10% and 0% activity left compared to TPMTwt (Fig. 4). In summary, the enzymatic activity of TPMTwt, TPMT p.Y240C and TPMT p.Y240S was in agreement with thermal stability data, reflected by the decreased enzymatic activity as a consequence of the lower thermal stability.

### The new SNP affect the SAM binding properties of TPMT

**Figure 5:**
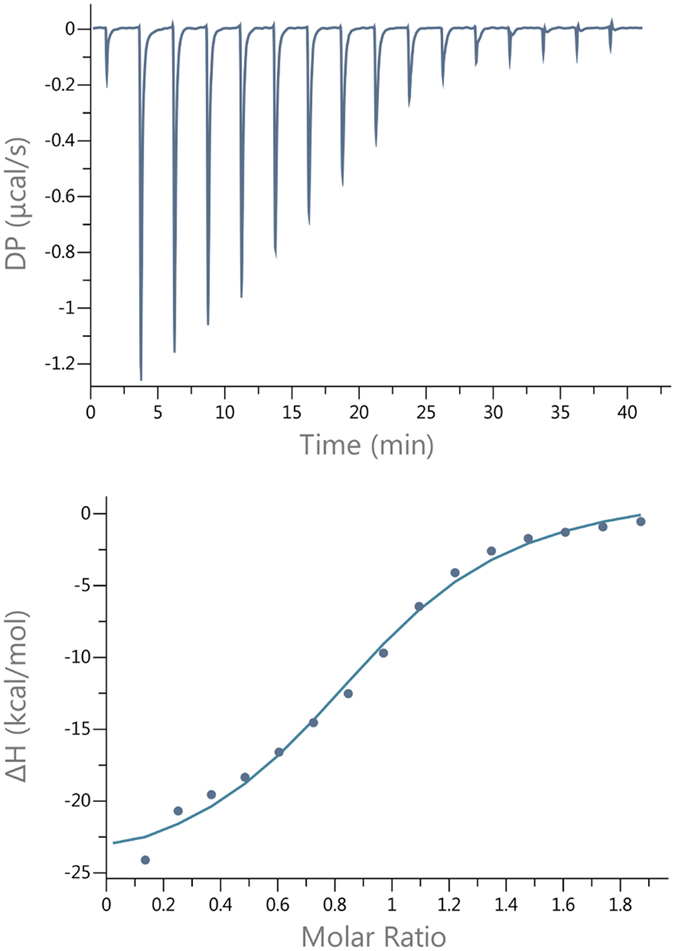
ITC data for the titration of SAM to TPMTwt. Raw data are shown in the upper panel together with fitted data in the lower panel.

To further characterize the biophysical properties of the proteins and in this case the binding affinities to the co-factor SAM, we performed isothermal titration calorimetry (ITC) on TPMTwt, TPMT p.Y240C and TPMT p.Y240S together with SAM. The initial results showed that both mutants displayed a weaker binding affinity towards SAM compared to TPMTwt. To date, a specific and reliable K_D_ value could only be determined between TPMTwt and SAM, showing a K_D_ of 2.7 μM (Fig. 5). However, by performing measurements on TPMT p.Y240C and TPMT p.Y240S at the same settings and conditions as for TPMTwt, we could conclude that the affinity towards SAM was weaker compared to TPMTwt (Fig. S1). The signal to noise ratio from the measurements were too high for TPMT p.Y240C and TPMT p.Y240S, thus giving to high uncertainties in K_D_ determination for these two variants. Anyhow, based on the fact that the signal was lowered when using the same settings and experimental conditions, we could still conclude that the affinity towards SAM were weaker for TPMT p.Y240C and TPMT p.Y240S compared to TPMTwt. This conclusion is also strengthened by the fact that CD results showed that higher amount of SAM were needed in order to stabilize TPMT p.Y240C and TPMT p.Y240S. Thus, the ITC measurements, together with the CD measurements, showed that SAM binding by TPMT p.Y240C and TPMT p.Y240S was affected by the SNPs c.719A > G and c.719A > C respectively.

### The protein structure network is severely weakened in p.Y240C and p.Y240S

**Figure 6:**
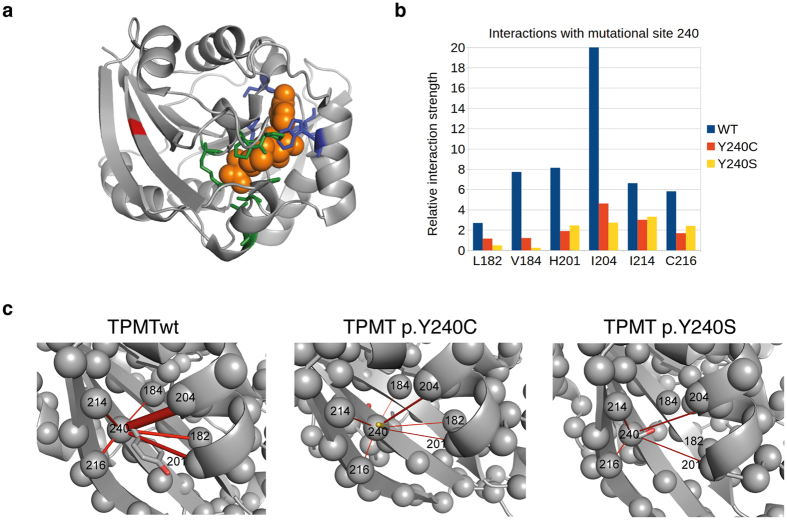
(**a**) Three-dimensional structure of human TPMT with the Y240 position in the β9-sheet marked in red. The bound coproduct of SAM, S-adenosylhomocysteine (SAH) is showed as spheres (orange) together with the SAH interacting residues (blue). Residues involved in binding the substrate 6-MP are showed in green. (**b**) Analysis of the relative interaction strength for positions interacting with position 240 from the MD simulations of WT, Y240C, and Y240S. (**c**) Relative interaction strengths visualized in the three-dimensional structure as red rods with a thickness proportional to the interaction strength for TPMTwt, TPMT p.Y240C and TPMT p.Y240S respectively. For clarity only the interactions with a relative strength larger than 1.0 are included.

To understand the molecular consequences of the mutations, molecular dynamics simulations starting structure from TPMTwt, TPMT p.Y240C, and TPMT p.Y240S were performed. In total, five independent 200 ns trajectories for each of the starting conformation were calculated. The resulting protein structure networks based on the last 150 ns of the trajectories were analyzed by representing the residue-residue contact network by a probabilistic graph model using the Protein Structure Network model in Wordom^31^. This analysis revealed that Tyr240 in the TPMTwt is involved in a strong network with the residues His182, Val184, His201, Ile204, Ile214, and Cys216 (Fig. 6b, c). This network was severely weakened in p.Y240C and even more so in p.Y240S, illustrated by the much lower relative interaction strengths (Fig. 6b, c). In particular, the interactions with Ile204 and His201 in helix α8 and the Val184 and Cys216 in the neighboring β-sheets (β7 and β8) were much less prevalent in any of the mutants compared to the TPMTwt. Val184 (β7) is close to the 6-MP substrate-binding site and the almost complete loss of interactions to that site in both mutants might directly destabilize the binding site.

## Discussion

In this study we addressed the question of whether SAM could stabilize TPMT and in addition we biophysically characterized a novel TPMT protein variant, TPMT p.Y240S together with the already known mutant at the same position TPMT p.Y240C (TPMT*3C)^13^,^14^,^15^. Although the newly discovered SNP c.719A > C seems to be rare and to this date, only found in one out of approximately 500 patients screened, comparison with TPMT p.Y240C (TPMT*3C), which is one of the most common alleles in both European and Asian populations are valuable for further understanding of the TPMT enzyme. The obtained T_m_ temperatures for TPMTwt and TPMT p.Y240C, 47.2 °C and 41.6 °C respectively, are in agreement with previous studies^30^ and we show that the novel protein variant, TPMT p.Y240S is even less stable than TPMP p.Y240C.

TPMT p.A154T/Y240C (TPMT*3A), which carries SNPs at position 240 as well as 154, results in very low expression of protein and no enzymatic activity, and forms aggregates *in vitro*^30^,^32^,^33^. In addition, TPMT p.A154T (TPMT*3B), which only carries SNP at 154, is not protected in rabbit reticulocyte lysate (RRL) degradation studies by the addition of SAM, while TPMT p.Y240C is protected^34^. Consequently, previously published results as well as the results presented here indicate that Tyr240 is important for TPMT stability and activity. In addition, the results from the Molecular Dynamics (MD) simulation show that Ile204 is one of the most connected residues in the protein network (Table S1) and the relative interaction strength between Tyr240 and Ile204 is lowered by the mutations at Tyr240 (Fig. 6b).

A sequence alignment display position 240 as strictly conserved and *in silico* test using various software display this position as deleterious for the protein. The SIFT analysis predicted the substitution at position 240 from Tyr to Cys as deleterious for the protein. In addition, PolyPhen-2 predicted both substitutions as probably damaging and both substitutions are predicted to interfere with protein function by intermediate probability by Align GVGD. Tyr240 is located in the central β-sheet core at the β9 stand (Fig. 6a) and interacts with residues in β7, β8, β9 and α8^35^. Mutation at position 240 has been suggested to affect both the hydrophobic core and intramolecular polar interactions^19^. Indeed, supported by the molecular dynamics simulation, both TPMT p.Y240C and TPMT p.Y240S lose polar interactions with neighboring β-sheets and the hydrophobic interactions with residues in α8. The loss of interactions was most severe for Y240S, which agrees with the thermal stability of the mutations.

The lowered thermal stability of TPMT p.Y240C and TPMT p.Y240S protein compared to TPMTwt protein was reflected in the enzymatic activity assay, where TPMT p.Y240C displayed very low activity and in the case for TPMT p.Y240S zero activity after 8 hours at 37 °C. This is not especially surprising, since the T_m_ in both cases are relatively close or almost 37 °C (41.6 and 36.7 °C, respectively). MD simulations of Catechol-O-methyl transferase (COMT) p.V108M suggest a larger amount of inactive protein at 37 °C^36^ and the lowered enzymatic activity may be explained by the lowered thermal stability seen for TPMT p.Y240C and TPMT p.Y240S, as well as COMT p.V108M^36^, resulting in less active protein molecules. Also, rabbit reticulocyte lysate (RRL) degradation studies on TPMT p.A154T/Y240C (TPMT*3A) and TPMT p.Y107D (TPMT*27) showed accelerated degradation of protein, with 38% and 48% remaining after 6 h, which is associated with lowered TPMT enzymatic activity in COS-1 cells^30^,^37^.

In a related study on COMT and the SNP resulting in the protein variant p.V108M, SAM was shown to increase the thermal stability for both the COMT p.V108M mutant and the wild-type enzyme. The increase in thermal stability was most pronounced for wild-type COMT, showing an increased ∆T_m_ by 5.7 °C compared to 3.5 °C for the p.V108M mutant^38^. In our studies, we could observe a small general increase in thermal stability on addition of 10-molar excess of SAM. The increased T_m_ was only significant for the TPMTwt protein. This is in agreement with ITC data, reflected by the higher affinity between TPMTwt and SAM compared to TPMT p.Y240C and TPMT p.Y240S. By adding SAM to 50-fold molar excess, the overall secondary structure was stabilized for all proteins (Fig. 3). The increase in thermal stability, by approximately 5 °C, is in agreement with the observed increase for COMT^38^. Interestingly, and in common for both TPMT and COMT, the binding site for SAM is not in direct contact with the p.Y240S (TPMT) or p.V108M (COMT) positions, with distances of approximately 16 Å and 17 Å respectively^38^. MD simulation of wild-type COMT and the p.V108M mutant indicate that introducing a methionine residue affects the solvent exposure at residue 108 and tertiary contacts as well as the structural ensemble, although the addition of SAM had no effect on the solvent accessibility of residue 108^36^. The greater solvent exposure at residue 108 altered the structural packing, dispersing through the structure to the SAM site 17 Å away^36^. Both COMT and TPMT share the basic Class I Methyltransferase fold^19^,^39^ and both COMT p.V108M, TPMT p.Y240C and TPMT p.Y240S are stabilized on SAM binding, despite the long distance between the polymorphic and SAM sites, it seems that TPMT may be affected in the same way as COMT. This is further emphasized by the fact that chemical shift perturbations and changes in backbone dynamics are seen in the bacterial orthologue of TPMT on binding the SAM analogue sinefungin^40^, indicating that binding of SAM can result in a structural effect not only at the SAM binding site^40^.

The possible stabilizing effect of SAM has been studied in cellular assays as well as *in vivo*. Since SAM is synthesized from L-methionine (Met), the intracellular concentration of Met is important for TPMT activity. Cells incubated in PBS buffer containing SAM or Met have higher TPMT activity compared to cells incubated in PBS only. The higher TPMT activity in SAM and Met treated cells is not a result of increased protein synthesis, instead the altered enzymatic activity is an effect of increased cellular stability of the TPMT protein^41^. This is in agreement with our thermal stability results, where TPMTwt as well as TPMT p.Y240C and TPMT p.Y240S were stabilized by the addition of SAM. Although our results show that both TPMTwt and TPMT p.Y240C can be stabilized by SAM, Milek *et al*. report a more pronounced Met depletion effect on TPMT p.Y240C protein levels^41^. In addition, our ITC results indicate that both TPMT p.Y240C and TPMT p.Y240S have substantially weaker affinity for SAM compared to TPMTwt. The higher molar excess of SAM needed to stabilize TPMT p.Y240C and TPMT p.Y240S protein further confirms the ITC results.

When discussing the stabilizing effect of SAM on TPMT in the context of 6-MP treatment. The importance of studying the influence of SAM on TPMT on a molecular level becomes even more clear by the fact that studies on cultured leukemic cells show that the cytotoxic effect of 6-MP can be reduced by the addition of exogenous SAM. The addition of exogenous SAM lowered the concentration of TGNs and methyl thioinosine monophosphate (MeTIMP)^23^, cytotoxic metabolites of 6-MP. In the same study, the addition of exogenous SAM reversed the 6-MP induced reduction of TPMT activity and protein levels. This emphasizes the question regarding possible co-treatment with SAM during 6-MP therapy, although we should bear in mind that this question is complex, due to the fact that SAM function as a co-factor to several methyltransferases, involved in various biological processes, which in turn can be influenced by the possible co-treatment with SAM. Thus, our study shows that changing only one amino acid can have a dramatic effect on TPMT stability and SAM binding properties. With this in mind, careful characterization of TPMT allelic variants, are especially important when further discussing which polymorphisms in the TPMT gene that effects thiopurine treatment.

Although several studies have discussed and evaluated the possible stabilizing effect of SAM both in cellular assays and *in vivo*, no biophysical studies have previously been performed on *in vitro* recombinant protein and demonstrated the stabilizing effect of SAM for fine-tuning the enzyme activity. More studies need to be done, evaluating the SAM binding on a more detailed level, since our study clearly shows that SAM increases the stability of TPMT and that changing only one amino acid can have a dramatic effect on TPMT stability and activity. In the future, it remains to be seen if the many reported decreased enzyme activity variants of TPMT are caused by changed affinity for the co-factor SAM and if lowered enzymatic activity could be overcome by supplementation with SAM or drugs affecting the folate metabolism.

## Material and Methods

### Ethical approval

The study was ethically approved by the Cluster Research Ethics Committee (KC/KE) of the Hong Kong Hospital Authority at Queen Elizabeth Hospital, Hong Kong SAR, China. Ethical approval project number KC/KE-14-0077/ER-1. Informed consent was obtained from the patient before collecting material for the study. All experiments were carried out in accordance with the approved and published guidelines.

### Summary of the patient with the novel SNP c.719A > C

The patient was referred to a rheumatology clinic and prescribed hydroxychloroquine and low dose azathioprine (25 mg daily) as an immunosuppressive agent. Blood was also collected for TPMT genotyping with the patient’s consent. The clinician was informed of the detection of a novel missense genetic variant c.719A > C which was predicted to be pathogenic by multiple in-silico tools. Azathioprine was then replaced with mycophenolate mofetil.

### Pathogenicity prediction

The TPMT protein sequence (P51580) was used in SIFT, PolyPhen-2 and Align GVGD pathogenicity prediction tools^28^,^29^,^42^,^43^. The SIFT sequence algorithm was used to search database UniRef90 2011 April (SWISS-PROT/TrEMBL), using a median sequence conservation of 3.00 and the removal of sequences with more than 90% identity to query. Score < 0.05 was considered deleterious. The multiple sequence alignment from SIFT analysis was further used in Align GVGD to predict the probability of the substitutions to interfere with protein function, where values between 0 < GV ≤ 61.3 and GD > 0 was functionally classified as deleterious 2. PolyPhen-2 score > 0.85 was classified as probably damaging.

### Analysis of the TPMT gene

Four variant alleles of the TPMT gene, TPMT*2 (Ala80Pro, rs1800462), TPMT*3A (Ala154Thr, rs1800460 and Tyr240Cys, rs1142345), TPMT*3B (Ala154Thr, rs1800460) and TPMT*3C (Tyr240Cys, rs1142345) at nucleotide positions c.238, c.460 and c.719 were detected using methods of polymerase chain reaction (PCR) followed by direct DNA sequencing. Genomic DNA was extracted from peripheral whole blood using a QIAamp DNA Blood Mini Kit according to the manufacturer’s instruction (Qiagen). The coding exons and the flanking introns of exons 5, 7 and 10 were amplified by PCR using sets of M13 tailed intronic primers (Table 2). The individual PCR reaction contained 100 ng DNA template, 25 μmol each of forward primer and reverse primer, 1x PCR buffer (Applied Biosystems), 37.5 mM MgCl_2_, 2.5 μM dNTP, and 1.25 U AmpliTaq Gold DNA polymerase (Applied Biosystems) in a total volume of 25 μL. PCR amplification was performed at a primer annealing temperature of 63 °C. Products were purified with Nucleospin^®^ Gel and PCR Clean-up Kit (Macherey-Nagel) and sequenced by using reagents from the BigDye^®^ Terminator v3.1 Cycle Sequencing Kit (Applied Biosystems) and M13 sequencing primers. Sequencing products were purified using a DyeEx^TM^ 2.0 Spin Kit (Qiagen), and the purified sequencing fragments were separated by capillary electrophoresis and detected by laser induced fluorescence on an ABI 3500 Genetic Analyzer (Applied Biosystems). Nucleotide changes and allelic variants were detected using the Mutation Surveyor software as well as manual inspection.

To exclude the possibility that the effect was from an additional SNP, the TPMT exon 2–10 were sequenced. DNA was isolated from whole blood using a Maxwell 16 Blood DNA Purification Kit (Promega) and amplified by PCR and the thermal profile: 95 °C for 15 min, 36 × (95 °C for 15 s, 55 °C for 15 s, 72 °C for 30 s), followed by a final extension at 72 °C for 10 min. The PCR conditions were as described previously^44^ except for exon 3 where the MgCl_2_ concentration was optimized to 4.0 mM. For primer sequences, see Table 2. The PCR amplicons were purified using an Illustra ExoProStar (GE Healthcare) and sequenced using a BigDye^®^ Terminator v3.1 Cycle Sequencing Kit (Applied Biosystems) and the capillary instrument 3500 Genetic Analyzer (Applied Biosystems).

### Pyrosequencing

DNA was isolated from whole blood using a Maxwell 16 Blood DNA Purification Kit (Promega). Exon 10 was amplified with PCR (for primers, see Table 2) and a thermal profile of 95 °C for 15 min, 45 × (95 °C for 15 s, 55 °C for 1.5 min, 72 °C for 30 s) followed by a final extension at 72 °C for 10 min, other conditions and reagents as described in^44^. Ten μl of PCR product was used for pyrosequencing using PyroMark96MD (Qiagen). The sequencing primer (Table 2) was designed to anneal adjacent to position c.719 and nucleotide dispension order CTGCATACT.

### Enzymatic activity in RBC

The enzymatic activity of TPMT SNP c.719A > C in RBCs was determined according to the previously published protocol^11^.

### Recombinant protein expression and purification

The different pET-28a vectors containing the human TPMTwt, TPMT p.Y240C, and TPMT p.Y240S constructs, were transformed into BL21(DE3) CodonPlus cells (Agilent) and cultured overnight at 37 °C in LB-Kanamycin (Kan)-Chloramphenicol (Cam) media. The overnight cultures were diluted and grown until OD_600_ = 0.8, induced with 1 mM isopropyl-β-D-1-thiogalactopyranoside (IPTG) and expressed overnight at 21 °C. Cells were harvested and resuspended in 20 mM Tris, 250 mM NaCl, 5 mM imidazole, 2 mM β-mercaptoethanol, 5% Glycerol, Complete^TM^ EDTA-free, protease inhibitor (Roche Applied Science), pH 8.0. Following sonication, the lysates were centrifuged at 18 000 g, 4 °C for 30 min.

The proteins were purified using Ni-NTA Superflow beads (Qiagen). The His-tag was cleaved off using biotinylated thrombin (Novagen), followed by capture of thrombin and His-tag onto streptavidin beads (Novagen) and Ni-NTA beads respectively. The proteins were further purified by gel filtration using a HiLoad Superdex 16/600 75 pg column (GE Healthcare).

### Circular dichroism (CD) measurements

All spectra were recorded on a Chirascan^TM^ spectrometer (Applied Photophysics) at 4 °C using 3 μM protein and 4 mm cuvette; 0.5 nm wavelength step and ten repeats at each wavelength. The T_m_ for each protein was recorded using 3 μM protein; 4 mm cuvette and temperature probe. The temperature range was set to 5–79 °C with steps of 2 °C in between each measurement and 60 sec. setting time. The α-helical unfolding was followed at 222 nm, using ten repeats. The long-term stability, was followed at 37 °C, measuring the CD signal at 222 nm every minute for 8 hours. Prior to all measurement, all samples were dialyzed against 20 mM KPi, 75 mM NaCl, 0.5 mM TCEP, 2% Glycerol, pH 7.3. All CD measurements were recorded in triplicate. The thermal denaturation temperatures were analyzed using the CDpal software^45^. In the software, data were normalized between 0 and 1, with 1 representing the value of unfolded protein, and the thermal denaturation temperatures were determined by plotting the fraction of α-helical unfolding at 222 nm as a function of temperature and fitting the data to the two-state denaturation model.

### Recombinant protein enzymatic activity assay

The enzymatic activity was measured after 0 h, 0.5 h and 8 h incubation at 37 °C using the SAMfluoro: SAM Methyltransferase Assay Kit (G-Bioscience). Protein samples and controls were prepared according to the protocol. Protein samples were dialyzed against 20 mM KPi, 75 mM NaCl, pH 7.3 and diluted to 10.5 μM to generate a final concentration of 0.46 μM in the assay. A concentration of 20 μM 6-MP was used as substrate. Protein samples and controls were measured in triplicate. The enzymatic activity was measured using an Infinite M1000 PRO plate reader (Tecan) with settings: excitation 535 nm, emission 590 nm, slit 10 nm and gain 72.

### Isothermal titration calorimetry (ITC)

The ITC experiments were performed using a PEAQ ITC instrument (Microcal). The samples were dialyzed against 20 mM KPi, 75 mM NaCl, 2 mM β-mercaptoethanol, 2% Glycerol, pH 7.3. The concentrations of TPMTwt, TPMT p.Y240C and TPMT p.Y240S in the cell were 27 μM, 29 μM and 25 μM respectively. A concentration of 250 μM SAM was used in the syringe. All measurements were performed at 25 °C, using reference power 7 μcal/s, 16 injections, 2.5 μl/injection and 150 s between each injection.

### Molecular dynamics (MD) simulations

The x-ray structure of the human TPMT (PDB ID: 2BZG) was used as the starting structure for TPMTwt and starting structures for TPMT p.Y240C and p.Y240S were constructed by mutating the amino acids in the wt structure *in silico*. All simulations were carried out with the Gromacs version 5.0.2^46^ package using the Amber99SB-ILDN force field^47^. Electrostatic interactions were evaluated using the Particle-Mesh-Ewald summation every step^48^. Replacing the hydrogens with virtual interaction sites enabled 4 fs time steps^49^. A 14 Å cutoff was used for both electrostatics and van der Waals interactions with neighbor lists updated every ten steps. Simulations were performed at 300 K using the velocity-rescaling algorithm^50^ and a constant pressure of 1.0 bar using the Berendsen pressure coupling algorithm^51^ time constant 1 ps and a compressibility of 4.5 10^−5^ bar^−1^.

Each starting conformation was first subjected to energy minimization using steepest descent followed by 0.5 ns with positional restraints on the protein using the LINCS algorithm^52^ allowing the water to equilibrate, without affecting the protein. This was followed by another 0.5 ns with weaker (10% of default) positional restraints, allowing the protein to slightly adapt to the surrounding water. Finally, the protein was simulated for 50 ns, allowing it to completely relax without any restraints (Fig. S2), before data were collected for 150 ns. This was repeated five times with different randomized starting velocities.

### Analysis of the molecular simulation

The networks of amino acid interactions in the molecular dynamics trajectories for each of the three starting structures were analyzed using the Protein Structure Network (PSN) module in the program Wordom^31^. The 3D structure of proteins are represented as a network, with amino acid side chains as nodes and non-covalent residue-residue interactions as links^53^. The following parameters were used in Wordom: distance cutoff, stable cutoff, hub contact cutoff and termini parameters at 4.5, 0.5, 3 and 0. The interaction strength with the mutation site was visualized in PyMol^54^.

### Statistical methods

A one-way ANOVA, using post-hoc Dunnett’s test, was performed on thermal denaturation data and long-time stability data. For enzymatic activity data, a two-way ANOVA was used. P ≤ 0.05 was considered as significant and statistical significance is plotted as P ≤ 0.05 (*), P ≤ 0.01 (**), P ≤ 0.001 (***). All statistical analyses were performed using the IBM, SPSS Statistics 23.0 (SPSS Inc, IBM).

## Additional Information

**How to cite this article:** Iu, Y. P. H *et al*. One amino acid makes a difference – Characterization of a new TPMT allele and the influence of SAM on TPMT stability. *Sci. Rep.* **7**, 46428; doi: 10.1038/srep46428 (2017).

**Publisher's note:** Springer Nature remains neutral with regard to jurisdictional claims in published maps and institutional affiliations.

## Supplementary Material

Supplementary Figures and Table

## Figures and Tables

**Table 1:** Time-dependent changes in CD signal in absence or presence of 10- or 50-fold molar excess of SAM.

Residual CD signal remaining after 8 hours measurements^a^ (%)			
Protein variant	−SAM	+SAM_10-fold_	+SAM_50-fold_
TPMTwt	85	97^***^	105^***^
TPMT p.Y240C	75	80	90^***^
TPMT p. Y240S	77	77	75

CD signal monitored at 222 nm. ^***^Significant level of one-way ANOVA: p ≤ 0.001.

^a^The percentage of CD signal remaining after 8 hours of measurements at 37 °C.

**Table 2:** PCR and sequencing primers.

Target Exon	Forward primer (5′ to 3′)	Reverse primer (5′ to 3′)
M13 tailed intronic primers^a^		
V	*TGTAAAACGACGGCCAGT*CTGCATGTTCTTTGAAACCCTATG	*CAGGAAACAGCTATGACC*CTGCGTGCTAAATAGGAACCATC
VII	*TGTAAAACGACGGCCAGT*GGGACGCTGCTCATCTTCT	*CAGGAAACAGCTATGACC*GCCTTACACCCAGGTCTCTG
X	*TGTAAAACGACGGCCAG*TAAGTGTTGGGATTACAGGTG	*CAGGAAACAGCTATGACC*TCCTCAAAAACATGTCAGTGTG
Sequencing exon 2 to 10		
II	AGCCTGGGGGATAGAGAGAG	GCAAACATTGCATAAAAGCAT
III	AGGTTTTCATTTAGTTCATCAAT	TTTTTGATAGAACATTTCTCTATTGT
IV	TACCACTGACTGGGTGTGTGTCTGA	CTCAATCCAGAAAGACTTCATACCTGTT
V	CCCTCTATTTAGTCATTTGAAAAC	ACTTTTGTGGGGATATGGAT
VI	GCCCTCTTTCCTTGACTATT	CACAGGAGGAAGAGAGTGAG
VII	TGTTGAAGTACCAGCATGCAC	AAAATTACTTACCATTTGCGATCA
VIII	CGAAAGTAACTTCTGGCTTC	GGCAACTGGTAAAAGAAAAA
IX	ATGCCACATCATCACCTATT	ACAGCATAAGTCCACAAACTT
X	CACCCAGCCAATTTTGAGTA	CACCCAGCCAATTTTGAGTA
Pyrosequencing		
X	Biotin-CCCAGCCAATTTTGAGTATT	CAATTCCTCAAAACATGTCA
	*Sequencing primer*	
	TTTACTTTTCTGTAAGTAGA	

^a^PCR primers with M13 tails in italic.
