# Supplementary material for: One amino acid makes a difference–Characterization of a new TPMT allele and the influence of SAM on TPMT stability
Source: Sci Rep. 2017 May 2;7:46428. doi: 10.1038/srep46428 (PMC5411961; doi:10.1038/srep46428)
Supplement: Supplementary Figures and Table [file srep46428-s1.pdf]

Supplemental Information

One amino acid makes a difference – Characterization of a new TPMT allele and the influence of SAM on TPMT stability

Authors

Iu Yan Ping Heidi[1,6], Sara Helander[2,6], Anna Zimdahl Kahlin[2], Cheung Chun Wah[1], Chi Chung Shek[1], Leung Moon Ho[3], Björn Wallner[4], Lars-Göran Mårtensson[5], Malin Lindqvist Appell[2*]

Affiliations

[1] Chemical Pathology Laboratory, Department of Pathology, Queen Elizabeth Hospital, Hong Kong SAR, China.

[2] Division of Drug Research, Department of Medical and Health Sciences, Linköping University, SE-581 85 Linköping, Sweden

[3] Department of Medicine, Queen Elizabeth Hospital, Hong Kong SAR, China.

[4] Division of Bioinformatics, Department of Physics, Chemistry and Biology, Linköping University, 58183 Linköping, Sweden

[5] Division of Chemistry, Department of Physics, Chemistry and Biology, Linköping University, SE-581 83 Linköping, Sweden.

[6] Co-first author

* Corresponding author: Malin Lindqvist Appell

e-mail: malin.lindqvist.appell@liu.se

Supplemental figures and tables

Supplementary figure 1

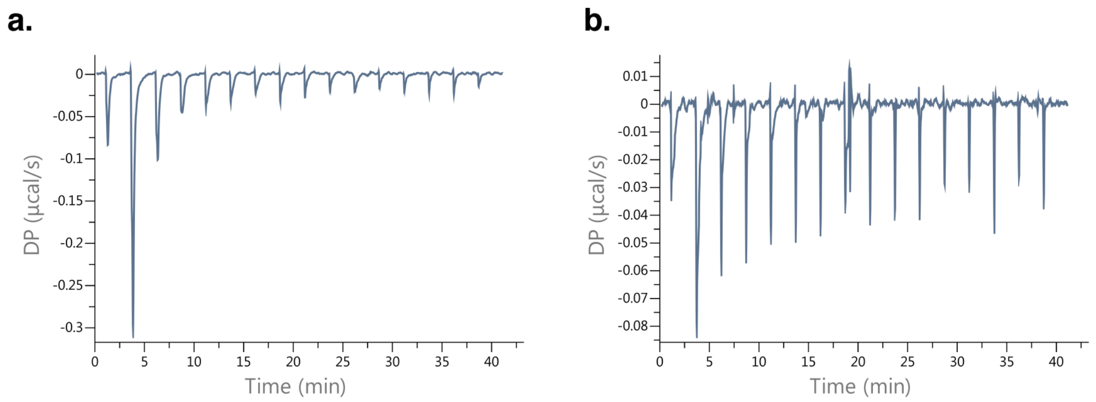

Figure S1. ITC data for the titration of SAM to TPMT p.Y240C (a) and TPMT p.Y240S (b). Due to the low signal to noise ratio data could not be fitted accurately and therefore only raw data are shown.

Supplementary figure 2

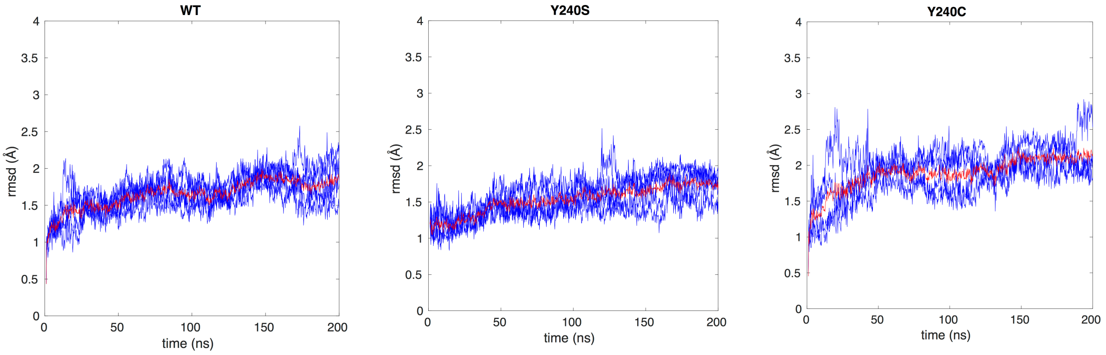

Figure S2. The root-mean-squared-deviation (rmsd) to the starting conformation for each of the five 200 ns trajectories for WT, Y240S, and Y240C (blue), the average highlighted in red.

Supplementary table 1

Table S1. The total interaction strength (Total Int), average interaction strength (Ave Int), and number of interacting residues (#Int) for the ten most interacting residues based on the protein network analysis of MD simulation.

Rank	chain:residue	Total Int	Ave Int	#Int
**1**	A:138	109.65	6.09	18
**2**	A:204	104.97	10.50	10
**3**	A:155	102.18	7.30	14
**4**	A:199	98.30	7.56	13
**5**	A:181	93.88	7.22	13
**6**	A:235	92.77	8.43	11
**7**	A:76	92.69	11.59	8
**8**	A:242	87.58	9.73	9
**9**	A:131	84.19	6.01	14
**10**	A:239	83.86	6.99	12
**11**	A:149	82.08	8.21	10
**12**	A:49	79.75	3.80	21
**13**	A:115	78.85	7.17	11
**14**	A:79	78.00	6.00	13
**15**	A:130	77.87	9.73	8
**16**	A:57	76.81	5.49	14
**17**	A:69	76.02	4.22	18
**18**	A:135	75.88	3.79	20
**19**	A:53	74.71	5.75	13
**20**	A:182	74.04	8.23	9
**21**	A:184	73.98	9.25	8
**22**	A:150	73.47	5.25	14
**23**	A:166	72.93	3.47	21
**24**	A:174	72.10	6.01	12
**25**	A:143	72.08	6.01	12
**26**	A:170	68.70	3.43	20
**27**	A:148	68.39	5.26	13
**28**	A:112	67.85	6.78	10
**29**	A:66	66.86	5.57	12
**30**	A:86	66.77	7.42	9
**31**	A:128	66.11	6.61	10
**32**	A:185	66.02	4.40	15
**33**	A:173	63.79	6.38	10
**34**	A:180	63.12	5.26	12
**35**	A:214	62.44	7.81	8
**36**	A:163	60.66	3.37	18
**37**	A:120	60.56	12.11	5
**38**	A:89	59.85	5.44	11
**39**	A:65	59.23	8.46	7
**40**	A:146	58.93	5.89	10
**41**	A:72	58.72	4.52	13
**42**	A:158	57.23	4.09	14
**43**	A:99	56.30	4.33	13
**44**	A:208	56.03	4.31	13
**45**	A:96	55.73	5.07	11
**46**	A:63	55.65	6.18	9
**47**	A:207	55.41	6.16	9
**48**	A:230	53.84	2.69	20
**49**	A:154	53.76	4.89	11
**50**	A:118	53.72	5.37	10
**51**	A:29	53.33	1.62	33

**52**	A:48	52.46	2.91	18
**53**	A:156	52.29	5.23	10
**54**	A:56	52.28	3.27	16
**55**	A:26	52.04	2.37	22
**56**	A:232	52.00	5.20	10
**57**	A:240	51.64	5.16	10
**58**	A:87	50.64	7.23	7
**59**	A:141	50.35	5.04	10
**60**	A:217	49.73	4.97	10
**61**	A:227	48.96	4.08	12
**62**	A:100	47.45	5.27	9
**63**	A:241	47.26	4.30	11
**64**	A:78	47.03	3.14	15
**65**	A:121	46.23	5.78	8
**66**	A:67	44.62	4.96	9
**67**	A:203	43.18	3.32	13
**68**	A:46	43.10	2.69	16
**69**	A:187	42.33	4.23	10
**70**	A:24	41.53	1.98	21
**71**	A:152	41.13	2.06	20
**72**	A:237	41.07	2.74	15
**73**	A:178	40.10	4.01	10
**74**	A:84	39.21	4.90	8
**75**	A:77	38.80	2.98	13
**76**	A:192	38.80	2.98	13
**77**	A:91	38.54	2.75	14
**78**	A:151	37.28	3.73	10
**79**	A:82	35.95	3.99	9
**80**	A:119	35.24	7.05	5
**81**	A:33	35.03	2.06	17
**82**	A:80	34.52	4.32	8
**83**	A:68	33.86	3.76	9
**84**	A:52	33.85	2.60	13
**85**	A:107	33.11	6.62	5
**86**	A:179	32.85	2.99	11
**87**	A:167	32.45	3.24	10
**88**	A:32	32.15	1.53	21
**89**	A:171	31.34	2.24	14
**90**	A:211	31.30	3.13	10
**91**	A:196	29.74	1.65	18
**92**	A:75	29.40	2.67	11
**93**	A:133	28.61	3.58	8
**94**	A:105	28.57	3.17	9
**95**	A:90	28.31	2.18	13
**96**	A:109	28.20	7.05	4
**97**	A:40	28.19	1.04	27
**98**	A:38	27.29	1.09	25
**99**	A:74	27.23	2.09	13
**100**	A:169	27.04	2.70	10
**101**	A:97	26.87	3.36	8
**102**	A:41	26.65	1.40	19
**103**	A:191	26.55	2.66	10
**104**	A:201	25.77	2.58	10
**105**	A:64	25.59	3.20	8
**106**	A:93	25.44	3.18	8
**107**	A:157	25.31	1.69	15
**108**	A:102	25.25	2.81	9
**109**	A:220	24.98	2.08	12
**110**	A:28	24.96	1.92	13
**111**	A:98	24.57	2.23	11
**112**	A:103	23.61	1.97	12

**113**	A:183	23.18	2.90	8
**114**	A:197	23.08	1.44	16
**115**	A:159	22.06	1.47	15
**116**	A:70	21.74	3.11	7
**117**	A:198	20.96	1.31	16
**118**	A:137	20.82	2.08	10
**119**	A:122	20.09	4.02	5
**120**	A:222	19.88	2.21	9
**121**	A:81	19.46	3.89	5
**122**	A:136	19.05	1.27	15
**123**	A:30	18.89	1.57	12
**124**	A:50	18.77	2.09	9
**125**	A:147	18.63	2.33	8
**126**	A:140	18.61	1.69	11
**127**	A:110	18.47	3.08	6
**128**	A:189	18.39	2.63	7
**129**	A:238	18.16	1.82	10
**130**	A:212	18.00	2.00	9
**131**	A:160	17.50	1.17	15
**132**	A:139	17.36	1.58	11
**133**	A:219	17.31	2.88	6
**134**	A:19	17.08	0.78	22
**135**	A:132	16.61	2.37	7
**136**	A:18	16.57	0.66	25
**137**	A:73	16.38	1.49	11
**138**	A:47	15.84	0.93	17
**139**	A:223	15.72	2.25	7
**140**	A:60	15.58	1.20	13
**141**	A:236	15.47	2.21	7
**142**	A:244	15.41	1.71	9
**143**	A:206	14.85	1.49	10
**144**	A:162	14.43	0.96	15
**145**	A:195	14.09	0.67	21
**146**	A:42	14.05	0.88	16
**147**	A:216	13.88	3.47	4
**148**	A:114	13.73	2.29	6
**149**	A:134	13.70	1.71	8
**150**	A:218	13.55	1.51	9
**151**	A:129	13.51	2.70	5
**152**	A:55	13.27	2.21	6
**153**	A:226	13.24	0.78	17
**154**	A:164	13.10	1.31	10
**155**	A:188	12.17	2.43	5
**156**	A:224	12.09	1.01	12
**157**	A:17	12.06	0.43	28
**158**	A:39	11.86	0.70	17
**159**	A:228	11.79	2.36	5
**160**	A:177	11.75	2.35	5
**161**	A:37	11.72	0.49	24
**162**	A:92	11.58	1.45	8
**163**	A:54	11.47	1.64	7
**164**	A:94	11.43	1.63	7
**165**	A:25	11.37	0.95	12
**166**	A:123	10.95	1.83	6
**167**	A:233	10.56	2.11	5
**168**	A:243	10.40	1.73	6
**169**	A:205	9.90	1.41	7
**170**	A:127	9.90	1.10	9
**171**	A:34	9.73	1.08	9
**172**	A:22	9.73	0.57	17
**173**	A:215	9.38	1.56	6

**174**	A:31	8.89	0.81	11
**175**	A:168	8.69	1.24	7
**176**	A:43	8.67	0.67	13
**177**	A:20	8.23	0.41	20
**178**	A:186	7.38	0.92	8
**179**	A:23	7.28	0.49	15
**180**	A:35	7.10	0.59	12
**181**	A:225	6.99	1.75	4
**182**	A:51	6.71	1.12	6
**183**	A:213	6.21	3.10	2
**184**	A:190	6.10	1.02	6
**185**	A:193	5.90	0.42	14
**186**	A:200	5.63	0.80	7
**187**	A:44	5.04	0.56	9
**188**	A:21	4.70	0.25	19
**189**	A:165	4.44	0.56	8
**190**	A:124	4.27	1.42	3
**191**	A:101	3.93	0.98	4
**192**	A:106	3.88	1.94	2
**193**	A:142	3.57	0.40	9
**194**	A:58	3.35	0.26	13
**195**	A:221	2.97	0.49	6
**196**	A:27	2.91	0.22	13
**197**	A:85	2.72	1.36	2
**198**	A:245	2.68	0.38	7
**199**	A:202	2.33	0.39	6
**200**	A:172	2.05	0.41	5
**201**	A:229	1.39	0.15	9
**202**	A:108	1.25	0.63	2
**203**	A:210	1.20	0.15	8
**204**	A:234	1.18	0.24	5
**205**	A:125	0.88	0.29	3
**206**	A:116	0.80	0.16	5
**207**	A:145	0.71	0.10	7
**208**	A:111	0.50	0.25	2
**209**	A:61	0.36	0.12	3
**210**	A:176	0.11	0.03	4
**211**	A:113	0.02	0.00	4
**212**	A:104	0.02	0.01	2
**213**	A:95	0.00	0.00	0
**214**	A:88	0.00	0.00	0
**215**	A:83	0.00	0.00	0
**216**	A:71	0.00	0.00	0
**217**	A:62	0.00	0.00	0
**218**	A:59	0.00	0.00	0
**219**	A:45	0.00	0.00	0
**220**	A:36	0.00	0.00	0
**221**	A:231	0.00	0.00	0
**222**	A:209	0.00	0.00	0
**223**	A:194	0.00	0.00	0
**224**	A:175	0.00	0.00	0
**225**	A:161	0.00	0.00	0
**226**	A:153	0.00	0.00	0
**227**	A:144	0.00	0.00	0
**228**	A:126	0.00	0.00	0
**229**	A:117	0.00	0.00	0
